# Acute kidney injury in patients with primary nephrotic syndrome: influencing factors and coping strategies

**DOI:** 10.1186/s12882-022-02720-y

**Published:** 2022-03-05

**Authors:** Honghua Lu, Liping Xiao, Mengqi Song, Xiaolan Liu, Fang Wang

**Affiliations:** 1 Department of Nephrology, Ganzhou People’s Hospital, Ganzhou, 341000 Jiangxi China; 2 Intensive Care Unit, Ganzhou People’s Hospital, No. 16, Meiguan Avenue, Zhanggong District, Ganzhou City, 341000 Jiangxi Province China

**Keywords:** Acute kidney injury, Primary nephrotic syndrome, Factors, Treatment, Nursing, Care

## Abstract

**Background:**

Acute kidney injury (AKI) is a frequent and serious complication in patients with primary nephrotic syndrome (PNS). We aimed to evaluate the influencing factors of AKI in patients with PNS, to provide implications for the clinical management and nursing care of patients with PNS.

**Methods:**

PNS patients who were treated in the Department of Nephrology in our hospital from January 1, 2020 to July 31, 2021 were included. The clinical characteristics and pathological type of PNS patients were evaluated. Pearson correlation and Logistic regression analysis were performed to analyze the related risk factors of AKI in patients with PNS.

**Results:**

A total of 328 patients with PNS were included, the incidence of AKI in PNS patients was 28.05%. Pearson correlation analysis showed that diabetes(*r* = 0.688), pulmonary infection (*r* = 0.614), albumin (*r* = 0.779), serum creatinine (*r* = 0.617), uric acid (*r* = 0.522), blood urea nitrogen (*r* = 0.616), renal tubular casts (*r* = 0.707) were correlated with AKI in PNS patients (all *P* < 0.05). Logistic regression analysis indicated that diabetes (OR2.908, 95%CI1.844 ~ 4.231), pulmonary infection(OR3.755, 95%CI2.831 ~ 4.987), albumin ≤ 24 g/L (OR1.923, 95%CI1.214 ~ 2.355), serum creatinine ≥ 90 μmol/L (OR2.517, 95%CI2.074 ~ 3.182), blood urea nitrogen ≥ 6.5 mmol/L (OR1.686, 95%CI1.208 ~ 2.123), uric acid ≥ 390 μmol/L (OR2.755, 95%CI2.131 ~ 3.371), renal tubular casts(OR1.796, 95%CI1.216 ~ 2.208) were the independently influencing factors of AKI in PNS patients (all *P* < 0.05).

**Conclusions:**

AKI is common in PNS patients. Actively controlling diabetes and pulmonary infection, strengthening nutrition support and renal function monitoring are essential to reduce the occurrence of AKI in PNS patients.

## Background

Acute kidney injury (AKI) is a common yet very serious complication in patients with primary nephrotic syndrome(PNS). Studies [1, 2] have shown that the incidence of AKI in children with PNS ranges from 1.28% to 38.26%, while the incidence of AKI in adults can be up to 44.9%. Because the previous criteria for diagnosing acute renal failure missed some patients in the early stage of AKI according to the guidelines of the Kidney Disease Improving Global Outcomes (KDIGO) [3], the actual incidence of AKI secondary to PNS may be much higher. Once AKI occurs, it can not only increase the length of hospital stay, medical expenses and death risk, but also delay the time to complete remission of nephrotic syndrome [4, 5]. Additionally, AKI is also an independent risk factor that causes nephrotic syndrome to progress to chronic kidney disease [6]. Therefore, the early identification and prevention of AKI is essential to the prognosis of PNS patients.

Currently, the mechanism of secondary AKI in patients with PNS is not completely clear. At present, it is believed that the occurrence of AKI may be related to intrarenal ischemia, renal interstitial edema, glomerular lesions, renal tubular necrosis, drug-related interstitial nephritis, etc. [7, 8] However, most of the reported studies are focused on the adult population, the clinical features and common pathological types of children with PNS are very different from those of adults with PNS. There are few reports on the relationship between the risk of adult AKI with PNS and changes in the pathological characteristics of the kidneys [9]. Therefore, this study retrospectively analyzed the clinicopathological characteristics of patients with PNS in our hospital, we aimed to analyze the influencing factors of AKI in patients with PNS, to provide evidences for the clinical management and nursing care of patients with PNS.

## Methods

In this study, all methods were performed in accordance with the relevant guidelines and regulations. Our study protocol had been checked and verified by the ethics committee of Ganzhou people's Hospital (approval number: E100945c), and written informed consent was obtained from all the included patients.

### Study population

We selected PNS patients who were treated in the Department of Nephrology in our hospital from January 1, 2020 to July 31, 2021. The inclusion criteria were as following: adult patients ≥ 18 years of age; patients who had been diagnosed with PNS after pathological diagnosis; Patients who were informed and agreed to participate in this study. patients were excluded for the following criteria: pregnant women; patients with malignant tumors; patients without a clear pathological type by renal biopsy; patients with secondary nephrotic syndrome; patients with missing clinical data.

### The diagnostic criteria of PNS

The diagnostic criteria for PNS [10] were as follows: (1). The patient had a large amount of proteinuria, with a quantitative urine protein > 3.5 g/L; (2) hypoalbuminemia, with plasma protein lower than 30 g/L; (3) High edema; (4) Hyperlipidemia. Among them, items 1, 2 were necessary for PNS diagnosis. We excluded secondary nephrotic syndrome caused by Henoch-Schonlein purpura, systemic lupus erythematosus, hepatitis B-related nephropathy and other diseases.

### Diagnostic criteria of AKI

The diagnosis of AKI referred to the relevant KDIGO guidelines [11]. AKI was diagnosed if any of the following criteria were met. (1) The absolute value of serum creatinine (Scr) increase within 48 h > 26.5 μmol/L (2) The Scr increase within 7 days > 1.5 times the baseline value; (3) Hourly urine output < 0.5 ml/kg, and lasts for more than 6 h. The baseline value of Scr was defined as the minimum value three months before admission. If it was not available, the minimum value during the patient's hospital stay was taken.

### Data collection

Two authors independently collected data from the medical records, any patients with missing information were excluded. We collected and organized the data of all PNS patients with a uniformly designed form, and the following data were collected: age; gender; hypertension; diabetes; pulmonary infection, which was diagnosed according to the diagnostic criteria [12] in China: all infections were diagnosed by bacterial culture and laboratory examination, body temperature ≥ 38 ℃, white blood cell ≥ 10.0 × 10^9^/L, X-ray showed lung inflammation change; related laboratory results at admission, including 24 h urine protein, albumin, serum creatinine, uric acid, blood urea nitrogen, total cholesterol, triglyceride, hemoglobin, D-dimer, fibrin degradation product, oliguria, polyserositis, proton pump inhibitor use, angiotensin converting enzyme use, diuretic use, antiplatelet drug use, pathological type and the pathological features of included PNS patients.

### Data analysis

We used SPSS 22. 0 Perform statistical processing on all collected data. Normally distributed measurement data were expressed in the form of mean ± standard deviation, and those that did not conform to the normal distribution were expressed in median (quartile). Persistent variables were compared between groups by t test, and categorical variables were compared between groups by chi-square test, Bonferroni correction was conducted to reduce the potential biases. Besides, we conducted the Pearson correlation analyses to identify the association of AKI and characteristics of PNS patients, Logistic regression model was used to analyze the related risk factors of AKI in patients with PNS. *P* < 0. 05 indicated that the group difference was statistically significant in this study.

## Results

A total of 328 patients with PNS were included, among whom 92 patients complicated by AKI, the incidence of AKI in PNS patients was 28.05% in this present study. As indicated in Table 1, there were significant differences in the gender, diabetes, pulmonary infection, albumin, serum creatinine, uric acid, blood urea nitrogen, triglyceride, oliguria, diuretic use, antiplatelet drug use between AKI and no AKI patients (all *P* < 0.05). No significant differences in the age, hypertension, 24 h urine protein, total cholesterol, hemoglobin, D-dimer, fibrin degradation product, polyserositis, proton pump inhibitor use, angiotensin converting enzyme use between AKI and no AKI patients were found (all *P* > 0.05).

**Table 1:** The characteristics of included PNS patients

Variable	AKI group (*n* = 92)	No AKI group (*n* = 236)	t/χ^2^	*P*
Age	54.07 ± 9.33	53.19 ± 10.14	9.471	0.086
Male/female	68/24	104/132	2.109	0.013
Hypertension	43(46.74%)	71(30.08%)	4.116	0.041
Diabetes	55(59.78%)	48(20.34%)	1.281	0.019
Pulmonary infection	51(55.43%)	23(9.75%)	1.366	0.007
24 h urine protein (mg/L)	5028.12 ± 1135.84	4897.09 ± 12.08	42.143	0.101
Albumin(g/L)	19.44 ± 5.02	24.17 ± 8.26	9.025	0.031
Serum creatinine (μmol/L)	106.38 ± 33.14	65.32 ± 19.42	14.226	0.001
Uric acid (μmol/L)	434.17 ± 108.55	341.09 ± 98.04	19.403	0.016
Blood urea nitrogen(mmol/L)	8.18 ± 3.15	4.75 ± 2.11	2.174	0.002
Total cholesterol (mmol/L)	8.59 ± 3.76	8.47 ± 3.88	2.105	0.116
Triglyceride (mmol/L)	2.39 ± 1.19	1.87 ± 1.02	1.124	0.018
Hemoglobin (g/L)	145.22 ± 28.17	146.19 ± 30.05	19.274	0.121
D-dimer (mg/ml)	1.28 ± 0.89	0.91 ± 0.36	1.905	0.104
Fibrin degradation product (μg/ml)	4.13 ± 1.77	3.09 ± 1.64	1.975	0.069
Oliguria	11(11.96%)	7(2.97%)	1.166	0.047
Polyserositis	81(88.04%)	196(83.05%)	2.042	0.064
Proton pump inhibitor use	88(95.65%)	220(93.22%)	2.132	0.074
Angiotensin converting enzyme use	29(31.52%)	71(30.08%)	1.914	0.101
Diuretic use	82(89.13%)	144(61.02%)	2.167	0.038
Antiplatelet drug use	75(81.52%)	103(43.64%)	1.115	0.013
Pathological type			4.007	0.129
Minor glomerular abnormalities	27(29.35%)	78(33.05%)		
Membranous nephropathy	17(18.48%)	40(16.95%)		
IgA nephropathy	18(19.57%)	45(19.07%)		
Non-IgA mesangial proliferative glomerulonephritis	13(14.13%)	29(19.29%)		
Focal segmental glomerulonephritis	12(13.04%)	24(10.17%)		
Membranoproliferative glomerulonephritis	2(2.17%)	5(2.12%)		
C1q nephropathy	2(2.17%)	3(1.27%)		
Other	1(1.09%)	2(0.85%)		

### The comparisons of pathological features

As presented in Table 2, the renal tubular casts of AKI patients were significantly more than that of no AKI patients (*P* = 0.012), there were no significant differences in the Glomerular sclerosis, parietal cytopathic lesions, podocyte lesion score, basal membrane lesion, capillary plexus lesion, mesangial proliferation, tubular atrophy, swelling of epithelial cells, vacuolation of epithelial cells, interstitial fibrosis and interstitial inflammatory infiltration between AKI and no AKI patients (all *P* > 0.05).

**Table 2:** The pathological features of included PNS patients

Variable	AKI group (*n* = 92)	No AKI group (*n* = 236)	t/χ^2^	*P*
Glomerular sclerosis	49(53.26%)	122(51.59%)	2.042	0.105
Parietal cytopathic lesions	9(9.78%)	20(8.47%)	1.127	0.092
Podocyte lesion score			4.884	0.178
0	28(30.43%)	70(29.66%)		
1	35(38.04%)	93(39.41%)		
≥ 2	29(31.53%)	73(30.93%)		
Basal membrane lesion	31(33.70%)	77(32.63%)	1.028	0.097
Capillary plexus lesion	40(43.48%)	92(38.98%)	2.144	0.131
Mesangial proliferation	89(96.74%)	222(94.07%)	5.102	0.077
Tubular atrophy			2.196	0.101
No atrophy	25(27.17%)	62(26.27%)		
< 5%	31(33.70%)	80(33.90%)		
≥ 5%	36(39.13%)	94(39.83%)		
Renal tubular casts	81(88.04%)	156(66.10%)	1.947	0.012
Swelling of epithelial cells	61(66.30%)	139(58.90%)	3.788	0.056
Vacuolation of epithelial cells	17(18.48%)	38(16.0%)	1.664	0.091
Interstitial fibrosis			4.107	0.106
No fibrosis	26(28.26%)	62(26.27%)		
< 5%	28(30.43%)	74(31.36%)		
≥ 5%	38(41.30%)	100(42.37%)		
Interstitial inflammatory infiltration			2.082	0.097
No infiltration	41(44.57%)	108(45.76%)		
< 5%	37(40.22%)	98(41.53%)		
≥ 5%	14(15.21%)	30(12.71%)		

### Pearson correlation analysis

As indicated in Table 3, Pearson correlation analysis showed that diabetes (*r* = 0.688), pulmonary infection (*r* = 0.614), albumin (*r* = 0.779), serum creatinine (*r* = 0.617), uric acid (*r* = 0.522), blood urea nitrogen (*r* = 0.616), renal tubular casts (*r* = 0.707) were correlated with AKI in PNS patients (all *P* < 0.05).

**Table 3:** Pearson correlation analysis of AKI and characteristics

Variables	r	p
Age	0.123	0.085
gender	0.118	0.071
Hypertension	0.204	0.112
Diabetes	0.688	0.018
Pulmonary infection	0.614	0.021
24 h urine protein (mg/L)	0.126	0.185
Albumin(g/L)	0.779	0.002
Serum creatinine (μmol/L)	0.617	0.013
Uric acid (μmol/L)	0.522	0.024
Blood urea nitrogen(mmol/L)	0.616	0.018
Total cholesterol (mmol/L)	0.261	0.092
Triglyceride (mmol/L)	0.201	0.099
Hemoglobin (g/L)	0.134	0.109
D-dimer (mg/ml)	0.284	0.067
Fibrin degradation product (μg/ml)	0.169	0.081
Oliguria	0.112	0.098
Polyserositis	0.204	0.115
Proton pump inhibitor use	0.109	0.094
Angiotensin converting enzyme use	0.231	0.077
Diuretic use	0.119	0.103
Antiplatelet drug use	0.202	0.079
Pathological type	0.114	0.093
Basal membrane lesion	0.246	0.065
Capillary plexus lesion	0.127	0.105
Mesangial proliferation	0.228	0.114
Tubular atrophy	0.109	0.084
Renal tubular casts	0.707	0.027
Swelling of epithelial cells	0.128	0.092
Vacuolation of epithelial cells	0.117	0.075
Interstitial fibrosis	0.263	0.091
Vacuolation of epithelial cells	0.281	0.094
Interstitial fibrosis	0.109	0.078
Interstitial inflammatory infiltration	0.113	0.107

### Logistic regression analysis

The variable assignments of multivariate logistic regression are shown in Table 4. As indicated in Table 5, Logistic regression analysis demonstrated that diabetes (OR2.908, 95%CI1.844 ~ 4.231), pulmonary infection (OR3.755, 95%CI2.831 ~ 4.987), albumin ≤ 24 g/L (OR1.923, 95%CI1.214 ~ 2.355), serum creatinine ≥ 90 μmol/L (OR2.517, 95%CI2.074 ~ 3.182), blood urea nitrogen ≥ 6.5 mmol/L (OR1.686, 95%CI1.208 ~ 2.123), uric acid ≥ 390 μmol/L (OR2.755, 95%CI2.131 ~ 3.371), renal tubular casts(OR1.796, 95%CI1.216 ~ 2.208) were the independently influencing factors of AKI in PNS patients (all *P* < 0.05).

**Table 4:** The variable assignments of multivariate logistic regression

Factors	Variables	Assignment
AKI	Y	No = 1, Yes = 2
Diabetes	X_1_	No = 1, Yes = 2
Pulmonary infection	X_2_	No = 1, Yes = 2
Albumin(g/L)	X_3_	> 24 = 1, ≤ 24 = 2
Serum creatinine (μmol/L)	X_4_	< 90 = 1, ≥ 90 = 2
Blood urea nitrogen(mmol/L)	X_5_	< 6.5 = 1, ≥ 6.5 = 2
Uric acid (μmol/L)	X_6_	< 390 = 1, ≥ 390 = 2
Renal tubular casts	X_7_	No = 1, Yes = 2

**Table 5:** Logistic regression analysis on the influencing factors of AKI in PNS patients

Variables	β	Wald	OR	95%CI	p
Diabetes	0.198	0.112	2.908	1.844 ~ 4.231	0.011
Pulmonary infection	0.145	0.114	3.755	2.831 ~ 4.987	0.004
Albumin ≤ 24 g/L	0.177	0.129	1.923	1.214 ~ 2.355	0.022
Serum creatinine ≥ 90 μmol/L	0.169	0.104	2.517	2.074 ~ 3.182	0.029
Blood urea nitrogen ≥ 6.5 mmol/L	0.184	0.121	1.686	1.208 ~ 2.123	0.013
Uric acid ≥ 390 μmol/L	0.174	0.116	2.755	2.131 ~ 3.371	0.041
Renal tubular casts	0.191	0.103	1.796	1.216 ~ 2.208	0.036

## Discussion

PNS is a group of common clinical syndromes, and its basic feature is massive proteinuria. Acute kidney injury is the most serious complication of PNS [13]. AKI is related to many factors, including renal interstitial edema, glomerular disease, hypoperfusion, renal tubular epithelial cells necrosis, renin–angiotensin–aldosterone system (RAAS) activation [14–16]. The incidence of AKI of PNS is relatively high. If it is not detected and treated in time, it will not only affect the prognosis of the patient, increase the pain and economic burden of the patient, and may be life-threatening in more severe cases [17, 18]. Therefore, it is very important to intervene the risk factors of AKI in a timely manner. We have found that the incidence of AKI in PNS patients is 28.05%, and diabetes, pulmonary infection, albumin ≤ 24 g/L, serum creatinine ≥ 90 μmol/L, blood urea nitrogen ≥ 6.5 mmol/L, uric acid ≥ 390 μmol/L, renal tubular casts are the independently influencing factors of AKI in PNS patients.

The results of this study have showed that the common pathological types of AKI are mild glomerular disease, Ig A nephropathy, and membranous nephropathy. Previous studies [5, 19] have shown that adult with mild glomerular disease is most susceptible to AKI, and the incidence of AKI is between 24.11% and 38.42%. Previous scholars [20, 21] have summarized 13 articles on mild glomerular disease associated with AKI published from 1993 to 2017, the results show that the incidence of AKI in mild glomerular disease patients is 33%, which is consistent with the results found in this study. At present, there are only sporadic reports on the relationship between specific renal pathological features and PNS secondary AKI. Studies [22, 23] have shown that renal tubular avascular necrosis in pathological damage is a risk factor for AKI. Pathological analysis of the kidneys in this study has showed that tubulin casts are an independent risk factor for AKI. Therefore, for those patients with protein casts, and their risk of AKI is significantly increased, early alerts on the development of AKI are needed in clinical setting.

Previous studies [24, 25] have shown that for every 10 g/L decrease in albumin level, the risk of AKI increases by 4.97 times. PNS patients are mostly accompanied by hypoalbuminemia, mainly due to the leakage of a large amount of proteinuria, and these proteinuria are closely related to renal damage, which can increase the occurrence of AKI [26]. The mechanism may be that urine protein can activate complement, promote chemotaxis and cytokines expression, causing endoplasmic reticulum stress, cell apoptosis, and damage to renal tubules [27]. In addition, patients with PNS often develop hyperuricemia due to relatively insufficient blood volume, diuretic use, abnormal renal function, and lipid metabolism disorders [28, 29]. Previous studies [30, 31] have reported that hyperuricemia can increase the risk of AKI in patients with PNS. This study further has confirmed that serum uric acid ≥ 390 μmol/L at admission of PNS patients is a risk factor for AKI. Hyperuricemia can activate the RASS and affect renal hemodynamics, leading to renal ischemia, and can also damage endothelial cells and renal interstitium [19, 32]. Besides, a sharp increase in blood uric acid can be formed in the renal tubules. Uric acid crystals block the renal tubules or compress the distal renal blood vessels, which can lead to the occurrence of AKI [33, 34].

Infections often occur in patients with PNS, which are related to the loss of cellular immunodeficiency, immunoglobulin Ig G and complement factors. This study shows that pulmonary infection is also a risk factor for AKI in patients with PNS. Most of the kidneys of PNS patients are in edema and ischemic state [35]. On this basis, infection may further aggravate renal ischemia and hypoxia, renal tubule damage, and affect kidney repair through immune inflammatory reaction, oxidative stress damage and other processes, and promote PNS patients AKI occurs [36, 37]. Previous studies [38–40] have reported that hypertension and diabetes are risk factors for the occurrence of AKI, but for PNS patients, the impact of hypertension and diabetes on AKI is currently unclear. We have found that diabetes is an independent risk factor for AKI in patients with PNS. Hyperglycemia can induce an increase in the synthesis of endothelin-1 by kidney cells, which can further aggravate renal tissue ischemia and increase the risk of AKI in patients with PNS. The independent risk factors based on the above-mentioned multivariate logistic regression analysis can provide references for early detection, early preventions and active treatment of AKI for patients with PNS, and have a good use value for clinical improvement of the prognosis of patients.

This study still has certain limitations merit consideration. Firstly, patients who have not undergone renal biopsy were excluded, resulting in a certain difference between the incidence of AKI and the actual situation. Secondly, the study was a single-center retrospective cohort study with a small sample size, and failed to build a predictive model for the occurrence of related AKI. The results of this study need to be further verified by a large sample of multi-center data in the future. Thirdly, this study only extracted relevant data during the patient's hospitalization, and did not perform long-term follow-up analysis. The choice of AKI treatment and the risk factors that affect the prognosis still need to be further studied.

## Conclusions

In summary, we have found that the incidence of AKI in PNS patients is 28.05%, and for PNS patients with diabetes, pulmonary infection, albumin ≤ 24 g/L, serum creatinine ≥ 90 μmol/L, blood urea nitrogen ≥ 6.5 mmol/L, uric acid ≥ 390 μmol/L, renal tubular casts, they may have higher risk of AKI. Patients with PNS should actively control pulmonary infections and diabetes, and correctly choose a reasonable treatment plan is vital to reduce the occurrence of AKI.

## Data Availability

All data generated or analyzed during this study are included in this published article. Xiaolan Liu (frcga0291737@163.com) should be contacted if someone wants to request the data.

## Abbreviations

AKI: Acute kidney injury
PNS: Primary nephrotic syndrome
KDIGO: Kidney Disease Improving Global Outcomes
Scr: Serum creatinine
RAAS: Renin–angiotensin–aldosterone system
